# N^6^-methyladenosine is required for efficient RNA synthesis of Ebola virus and other haemorrhagic fever viruses

**DOI:** 10.1080/22221751.2023.2223732

**Published:** 2023-06-21

**Authors:** Lisa Wendt, Matthew J. Pickin, Bianca S. Bodmer, Sven Reiche, Lucie Fénéant, Julia E. Hölper, Walter Fuchs, Allison Groseth, Thomas Hoenen

**Affiliations:** a Institute of Molecular Virology and Cell Biology, Friedrich-Loeffler-Institut, Greifswald – Insel Riems, Germany; b Institute of Novel and Emerging Infectious Diseases, Friedrich-Loeffler-Institut, Greifswald – Insel Riems, Germany; c Department of Experimental Animal Facilities and Biorisk Management, Friedrich-Loeffler-Institut, Greifswald – Insel Riems, Germany

**Keywords:** Ebola virus, Junín virus, Crimean-Congo haemorrhagic fever virus, filovirus, arenavirus, orthonairovirus, m^6^A, METTL3

## Abstract

N^6^-methyladenosine (m^6^A) is one of the most abundant modifications of cellular RNA, where it serves various functions. m^6^A methylation of many viral RNA species has also been described; however, little is known about the m^6^A epitranscriptome of haemorrhagic fever-causing viruses like Ebola virus (EBOV). Here, we analysed the importance of the methyltransferase METTL3 for the life cycle of this virus. We found that METTL3 interacts with the EBOV nucleoprotein and the transcriptional activator VP30 to support viral RNA synthesis, and that METTL3 is recruited into EBOV inclusions bodies, where viral RNA synthesis occurs. Analysis of the m^6^A methylation pattern of EBOV mRNAs showed that they are methylated by METTL3. Further studies revealed that METTL3 interaction with the viral nucleoprotein, as well as its importance for RNA synthesis and protein expression, is also observed for other haemorrhagic fever viruses such as Junín virus (JUNV) and Crimean-Congo haemorrhagic fever virus (CCHFV). The negative effects on viral RNA synthesis due to loss of m^6^A methylation are independent of innate immune sensing, as METTL3 knockout did not affect type I interferon induction in response to viral RNA synthesis or infection. Our results suggest a novel function for m^6^A that is conserved among diverse haemorrhagic fever-causing viruses (i.e. EBOV, JUNV and CCHFV), making METTL3 a promising target for broadly-acting antivirals.

## Introduction

The cellular epitranscriptome contains a plethora of modifications, many of which are still poorly understood. Among RNA modifications, N^6^-methyladenosine (m^6^A) is one of the most abundant and best studied. Methylation occurs co-transcriptionally and is mediated by the m^6^A writer complex, consisting of the methyltransferase dimer of METTL3 (the active methyltransferase) and METTL14 (the allosteric activator) [1–3]. Additionally, helper proteins like WTAP, RBM15, ZC3H13, VIRMA and HAKAI ensure the correct localization of the m^6^A writer complex and mediate the specificity of the methylation [4–6]. Proteins interacting with m^6^A, known as m^6^A readers, mediate the different functions of m^6^A. For example, YTHDC1 is associated with alternative splicing regulation and mRNA export, while YTHDF proteins, especially YTHDF2, promote mRNA instability and degradation [7–9]. The DRACH consensus sequence for m^6^A modification occurs very frequently in cellular mRNA, but few motifs are actually methylated and these mainly occur in 3’ untranslated regions (UTRs) and around stop codons [10,11].

m^6^A modification of viral RNA species has been observed for many viruses, albeit with very different biological outcomes [12]. Methylation of viral genomes appears disadvantageous for infectious flavivirus particle production or replication of Hepatitis B virus [13,14]. In contrast, for some negative-sense RNA viruses (NSVs) methylation of their RNA species is beneficial for their life cycles. For instance, loss of m^6^A in human metapneumovirus (HMPV) or vesicular stomatitis virus (VSV) RNAs leads to increased interferon beta (IFN-β) production, suggesting they use m^6^A to evade the innate immune response [15,16]. However, the role of m^6^A for haemorrhagic fever-causing NSVs, including the filovirus Ebola virus (EBOV), remains unknown.

Analysis of mass spectrometry (MS)-based EBOV interactome studies allows the identification of interactors common to specific host cell machineries [17–19]. Among the proteins identified in these studies are several associated with the m^6^A writer complex like WTAP and RBM15, as well as m^6^A reader proteins. Furthermore, we have previously identified both WTAP and RBM15 in a genome-wide siRNA screen of host factors supporting the EBOV life cycle [20]. Based on these data we hypothesized that m^6^A may play an important role in the EBOV life cycle, and assess the role of both m^6^A itself and METTL3, the essential methyltransferase in this pathway, in the EBOV life cycle. Further, we assess whether this function is conserved among other haemorrhagic fever-causing NSVs such as the arenavirus Junín virus (JUNV) and the orthonairovirus Crimean-Congo haemorrhagic fever virus (CCHFV).

## Material & methods

### Cells and viruses

Human hepatocarcinoma (Huh7) cells (kindly provided by Stephan Becker, Philipps University Marburg) and human embryonic kidney (HEK 293 T) cells (Collection of Cell Lines in Veterinary Medicine CCLV-RIE 1018), as well as the generated knockout (KO) cell lines were maintained in Dulbecco’s Modified Eagle’s medium (DMEM; Thermo Fisher Scientific) supplemented with 1x GlutaMAX (Thermo Fisher Scientific), penicillin (100 U/mL)/streptomycin (100 µg/mL) (Thermo Fisher Scientific) and 10% fetal bovine serum at 37 °C and 5% CO_2_.

The recombinant wildtype EBOV (rgEBOV), recombinant EBOV expressing firefly luciferase (rgEBOV-luc2) and recombinant EBOV expressing C-terminally flagHA tagged NP (rgEBOV-NP-flagHA) have been previously described [21–23]. All experiments involving infectious EBOV were performed in the BSL4 laboratory of the Friedrich-Loeffler-Institut following approved standard operating procedures.

### Co-immunoprecipitation of viral proteins

Co-immunoprecipitation was performed as previously described [24]. For further details see Supplemental methods.

### EBOV minigenome assays

The EBOV minigenome assays were performed as previously described [25]. For the complementation assay, cells were additionally transfected with pCAGGS-METTL3-ΔgRNA, which contains silent mutations in all guide RNA (gRNA)-binding sites. For further details see Supplemental methods.

### EBOV infection experiments

Parental, negCtrl and METTL3 KO cells were seeded in 12-well plates and infected 6 h later with rgEBOV-luc2 (MOI = 0.5). At 20 h post infection (hpi), cell culture supernatant was removed and cells were lysed for 10 min in 300 µl Glo Lysis Buffer (Promega). Supernatants were cleared of cell debris and 50 µl cell lysate was added to 50 µl CellTiter-Glo or Bright-Glo in a white 96-well plate. Luminescence was measured in a Tekan F200Pro and reporter activity was normalized against CellTiter-Glo values.

For the inhibitor assay, parental 293 T cells were infected with rgEBOV-luc2 (MOI = 0.5) and 4 and 24 hpi, cell culture supernatant was replaced by media containing either 30 µM STM2457 (in DMSO) or the corresponding amount of DMSO. Reporter activity was determinded 48 hpi as described above.

### Immunofluorescence analysis

Huh7 cells seeded in 8-chamber microscopy slides (ibidi) were infected with rgEBOV-NP-flagHA (MOI = 10). At 24 hpi, cells were fixed with 10% formalin and stained for METTL3 and VP30 using protein-specific antibodies and for NP using anti-HA antibodies as previously described [24]. For further details for the antibodies see Supplemental methods. Slides were analysed via confocal microscopy using a Leica SP5 with a 63x oil immersion objective (Leica Microsystems).

### miCLIP

NegCtrl and METTL3 KO cells were seeded in 6-well plates and infected one day later with rgEBOV (MOI = 1). 24 hpi, medium was exchanged against DMEM with 5% FCS containing 0.02 mg/ml Actinomycin D (Gibco), and 48 hpi supernatant was removed and cells were washed in 1 ml PBS per well. After centrifugation for 5 min at 1000 x g cell pellets were resuspended in 250 µl PBS and transferred to tubes containing 750 µl TRIzol (Thermo Fisher Scientific) and removed from the BSL4 laboratory. RNA was isolated according to the manufacturer’s instructions. mRNA was further isolated using the mRNA DIRECT Kit (Thermo Fisher Scientific) following the manufacturer’s instructions, and subsequently used for miCLIP analysis without fragmentation as described previously [26]. For further details see Supplemental methods.

### minION Sequencing

miCLIP samples were prepared for Nanopore sequencing using Direct cDNA Sequencing with Native Barcoding (Oxford Nanopore, cat. no. SQK-DCS109 and EXP-NBD104) following the manufacturer’s instructions. For further details see Supplemental methods.

### Minigenome assays of other NSVs

JUNV and CCHFV minigenome assays in 293 T cells were performed as previously described [27,28]. For complementation assays, cells were additionally transfected with pCAGGS-METTL3-ΔgRNA. For further details see Supplemental methods.

### RNA isolation and RT-qPCR

For determination of IFN-β mRNA levels in the different minigenome systems, minigenome assays were performed as described above, but instead of measuring reporter activity, RNA was isolated from cells using TRIzol following the manufacturer’s instructions. For determination of IFN-β levels in the context of an EBOV infection, parental, negCtrl and METTL3 KO cells were seeded in 12-well plates and infected one day later with rgEBOV (MOI = 0.5). At 24 hpi, cell culture supernatant was removed, cells were resuspended in 250 µl PBS and added to 750 µl TRIzol before removal from the BSL4 laboratory. RNA was isolated following the manufacturer’s instructions. DNA was digested (TURBO DNA-free kit; ThermoFisher Scientific) for 1 h prior to cDNA synthesis using RevertAid Reverse Transcriptase (Thermo Fisher Scientific) according to the manufacturer’s instructions using oligo(dT) primers. For subsequent qPCR analysis, 1 µl cDNA was used together with primers targeting either IFN-β or GAPDH (as a control) [29] and the PowerUp SYBR Green Master Mix (Thermo Fisher Scientific) following the manufacturer’s instructions.

## Results

### METTL3 interacts with EBOV proteins

**Figure 1.**
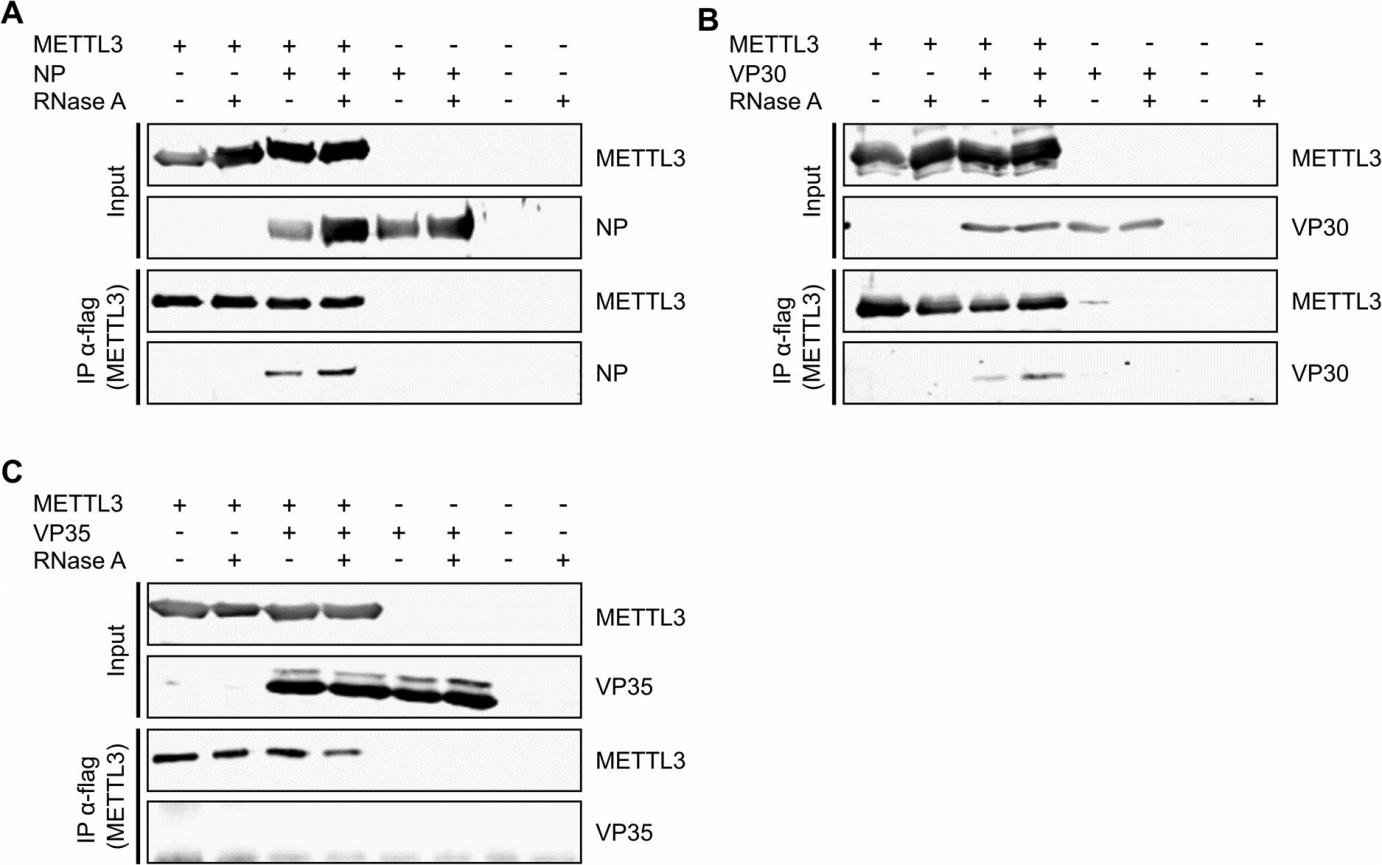
METTL3 interacts with EBOV proteins. 293 T cells were transfected with flagHA-METTL3 as well as expression plasmids for EBOV (a) NP, (b) VP30 or (c) VP35. Two days post transfection (dpt), cells were lysed and METTL3 was precipitated with flag-antibodies in the presence and absence of RNase A. Precipitates and input samples were analysed via SDS-PAGE and Western blot using anti-HA-antibodies for the detection of METTL3 as well as protein-specific antibodies for the detection of viral proteins. Representative results from two independent experiments are shown.

To assess whether the m^6^A writer METTL3 interacts directly with EBOV, we performed co-immunoprecipitations with flagHA-METTL3 and a subset of the EBOV ribonucleoprotein complex (RNP) proteins required for viral RNA synthesis: the nucleoprotein NP, which binds and encapsidates viral genomic RNA, as well as VP35 and VP30, RNA-binding proteins that act as the viral polymerase cofactor and transcriptional activator, respectively (Figure 1). All proteins were expressed and showed the expected molecular weight (Suppl. Figure 1). Using this approach, we were able to co-precipitate NP with METTL3, and this remained unchanged upon RNase treatment, showing that NP and METTL3 interact in an RNA-independent manner. Furthermore, we could co-precipitate EBOV VP30, but not VP35 with METTL3, and this interaction also remained unchanged after RNase treatment, demonstrating an RNA-independent METTL3-VP30 interaction.

### Localization of METTL3

**Figure 2.**
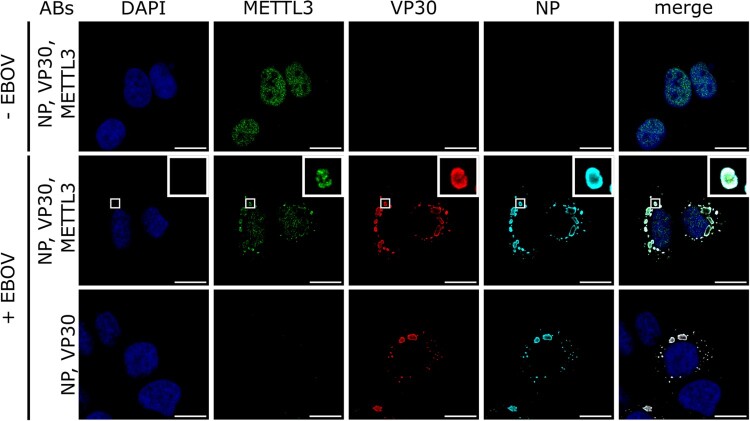
METTL3 localizes to EBOV inclusion bodies. Huh7 cells were infected with rgEBOV-NP-flagHA (MOI = 10) or remained uninfected. At 24 hpi, cells were fixed, permeabilized and stained for METTL3 and EBOV VP30 using protein-specific antibodies and for EBOV NP using an anti-HA antibody. Scale bars indicate 20 µm and insets show magnifications of indicated areas. Representative results from two independent experiments are shown.

As EBOV is known to replicate in cytoplasmic inclusion bodies [30], which are formed by NP and recruit VP30, cellular proteins interacting with these proteins would be expected to also be recruited to these structures. To assess whether METTL3 is localized in EBOV inclusion bodies, we performed immunofluorescence analyses of cells infected with a recombinant EBOV expressing a C-terminally flagHA-tagged NP [21] and stained the cells one day post infection for NP, VP30 and endogenous METTL3. Infected cells showed typical localization of NP and VP30 in inclusion bodies within the cytoplasm [30], and uninfected cells showed METTL3 staining predominantly in the nucleus, as previously described (Figure 2) [2]. When analysing the localization of METTL3 in EBOV-infected cells, METTL3 was still detectable in the nucleus, but we could also observe a strong co-localization with EBOV NP and VP30 in cytoplasmic inclusion bodies. However, the distribution pattern of METTL3 differed from that of NP and VP30, and was localized in punctae within the inclusion bodies. These results show that METTL3 is recruited into EBOV inclusion bodies.

### METTL3 is necessary for efficient viral RNA synthesis

**Figure 3.**
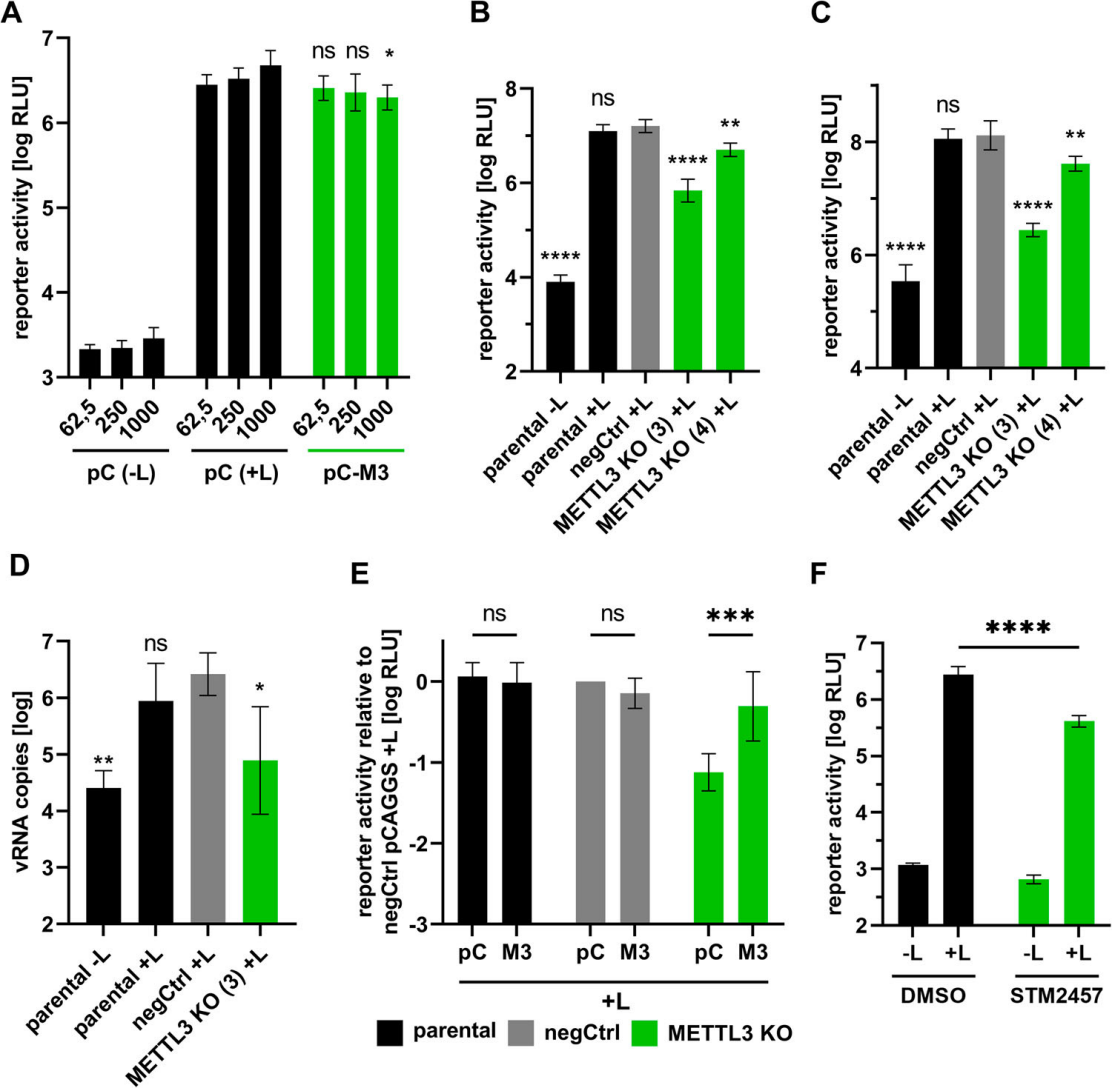
METTL3 is important for viral RNA synthesis. (A) Influence of METTL3 overexpression on EBOV RNA synthesis. 293 T cells were transfected with the plasmids for a replication-competent EBOV minigenome assay as well as increasing amounts of empty vector (pC) or a METTL3 expression plasmid (pC-M3). Amounts depicted in ng. As a negative control, L was omitted (-L). Two dpt, reporter activity was measured. (B, C) Influence of METTL3 KO on viral RNA synthesis and protein expression. 293 T parental, negative control (negCtrl) or METTL3 KO cells were transfected with the plasmids required for a (B) replication-competent or a (C) replication-deficient EBOV minigenome assay and two dpt, reporter activity was measured. (D) Importance of METTL3 for viral replication. Replication-competent minigenome assays were performed in parental, negCtrl or METTL3 KO cells and two dpt, RNA was isolated and the amount of genomic RNA (vRNA) was quantified via RT-qPCR. (E) Transcomplementation of METTL3 KO. Parental, negCtrl or METTL3 KO cell lines were transfected with all components for a replication-competent EBOV minigenome assay as well as either empty vector (pC) or pCAGGS-METTL3-ΔgRNA (M3). Reporter activity was determined two dpt. (F) Influence of METTL3 inhibition on EBOV RNA synthesis and protein expression. 293 T parental cells were transfected with all components for a replication-competent EBOV minigenome assay. 4 hpt and 24 hpt cells were treated with 30 µM STM2457 or DMSO. 48 hpt cells were harvested and reporter activity was determined. Means and standard deviations of four biological replicates from at least two independent experiments are shown. Asterisks indicate *p* values from one-way ANOVA with Sidak’s multiple comparisons test (*: *p* ≤ 0.05; **: *p* ≤ 0.01; ***: *p* ≤ 0.001; ****: *p* ≤ 0.0001; ns: *p* ≥ 0.05). Statistical significance was calculated compared to (A) the respective pC (+L) sample, (B-D) negCtrl (+ L) or (E) between the respective pC and M3 samples.

To evaluate whether the identified interactions have a functional relevance for the EBOV life cycle, and METTL3 plays a functional role in inclusion bodies, we first assessed whether overexpression of METTL3 influences viral RNA synthesis and/or protein expression by using classical, replication-competent minigenome assays, which model these viral processes (Figure 3A). Low amounts of METTL3 had no effect on reporter activity (reflecting viral RNA synthesis and protein expression), but at the highest amounts of the METTL3 expression plasmid we observed a small (2.4 fold) decrease in reporter activity compared to the empty vector control. To further analyse the role of METTL3 for viral RNA synthesis, we generated clonal 293 T METTL3 knockout (KO) cell lines using CRISPR/Cas9-based genomic editing. Western blotting confirmed the lack of detectable METTL3 in the two cell clones used in this study (Suppl. Fig. 2A). In parallel, a clonal negative control cell line (negCtrl) was produced using a scrambled gRNA with no known targets in the human genome. In order to confirm biallelic KOs in both generated KO cell lines, genomic DNA was isolated and cloned in order to allow sequencing of the *METTL3* gene. Sequencing of 5 and 15 clones, respectively, confirmed a biallelic KO for both cell lines, as they both showed deletions in exon 3 leading to frame shifts and premature stop codons (Suppl. Fig. 2B).

Using these cell lines we then analysed the role of METTL3 for EBOV RNA synthesis with different life cycle modelling systems. First, we assessed the importance of METTL3 for viral RNA synthesis and/or protein expression using a replication-competent minigenome system, which models these steps in the viral life cycle (Suppl. methods). Here, reporter activity in the negCtrl cell line was comparable to the parental cell line, while in the METTL3 KO cell lines a strong reduction in reporter activity was observed (Figure 3B), indicating that METTL3 is important for EBOV RNA synthesis and/or protein expression. To further dissect the RNA synthesis process, we also performed replication-deficient minigenome assays in these cells. This assay uses a minigenome with a deletion in the antigenomic replication promotor, which allows modelling of viral RNA transcription and protein expression independent of viral RNA replication [25,31]. Using this assay, we observed a strong reduction in reporter activity in the KO cell lines (Figure 3C), indicating that METTL3 is important for viral transcription and/or protein expression. To also assess the role of METTL3 for viral replication directly, we analysed minigenomic vRNA levels in a replication-competent minigenome assay by RT-qPCR. Both parental and negCtrl cell lines showed robust vRNA levels, while vRNA levels in the METTL3 KO cell line were reduced (Figure 3D), indicating that METTL3 is also important for viral RNA replication. To confirm that METTL3 is responsible for this effect, we complemented the METTL3 KO by transfecting pCAGGS-METTL3-ΔgRNA (a METTL3 expression plasmid with mutations in the *METTL3* gRNA target sites) in a replication-competent minigenome assay. Using this approach, we could restore reporter activity (Figure 3E), indicating that the observed effects are due to the METTL3 KO and not non-specific effects in the selected cell clones. To further confirm the specificity of the observed effect, we performed replication-competent EBOV minigenome assays in presence of the METTL3 inhibitor STM2457 (Figure 3F) [32]. Similar to the effects observed upon METTL3 KO, inhibition of METTL3 led to a strong reduction in reporter activity when compared to DMSO-treated cells, confirming that METTL3 is important for efficient EBOV RNA synthesis.

### m^6^A writer METTL3 is also important during an EBOV infection

**Figure 4.**
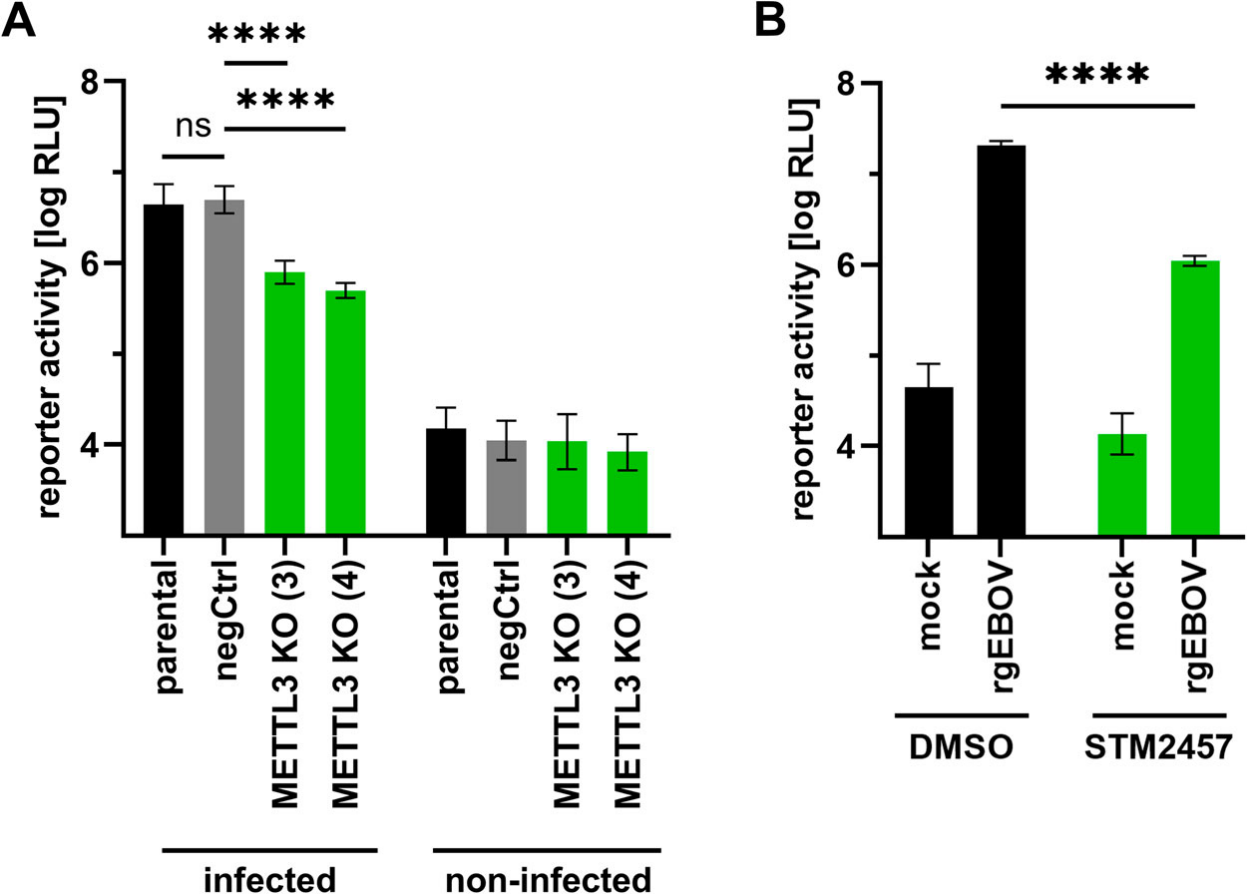
METTL3 is important for EBOV infection. (A) Parental, negCtrl and METTL3 KO 293 T cells were infected with a luciferase-expressing EBOV (MOI = 0.5). At 20 hpi, cells were lysed and reporter activity was determined. Normalized reporter activities from five biological replicates from three independent experiments are shown. (B) Parental 293 T cells were infected with a luciferase-expressing EBOV (MOI = 0.5). 4 and 24 hpi cells were either treated with 30 µM STM2457 or the equivalent amount of DMSO. Reporter activity was determined 48 hpi. Shown are normalized reporter activities from four biological replicates from two independent experiments. Asterisks indicate *p* values from one-way ANOVA with Sidak’s multiple comparisons test (****: *p* ≤ 0.0001; ns: *p* ≥ 0.05).

After having demonstrated the importance of METTL3 for viral RNA synthesis using life cycle modelling systems, we next confirmed these data in the context of an infection with authentic EBOV. Parental, negCtrl and METTL3 KO 293 T cell lines were infected with recombinant EBOV expressing firefly luciferase from an additional transcriptional unit [22], and reporter activity was measured one day post infection. Using this approach, we observed a reduction in luciferase activity (again reflecting viral RNA synthesis and protein expression) in the METTL3 KO cell lines in comparison to parental and negCtrl cells (Figure 4), similar to what we had observed in the minigenome assay (Figure 3B). Furthermore, inhibition of METTL3 by STM2457 also led to a strong reduction in reporter activity, confirming the results from the KO cell lines (Figure 4B).

### Mapping of m^6^A modifications in EBOV mRNA

**Figure 5.**
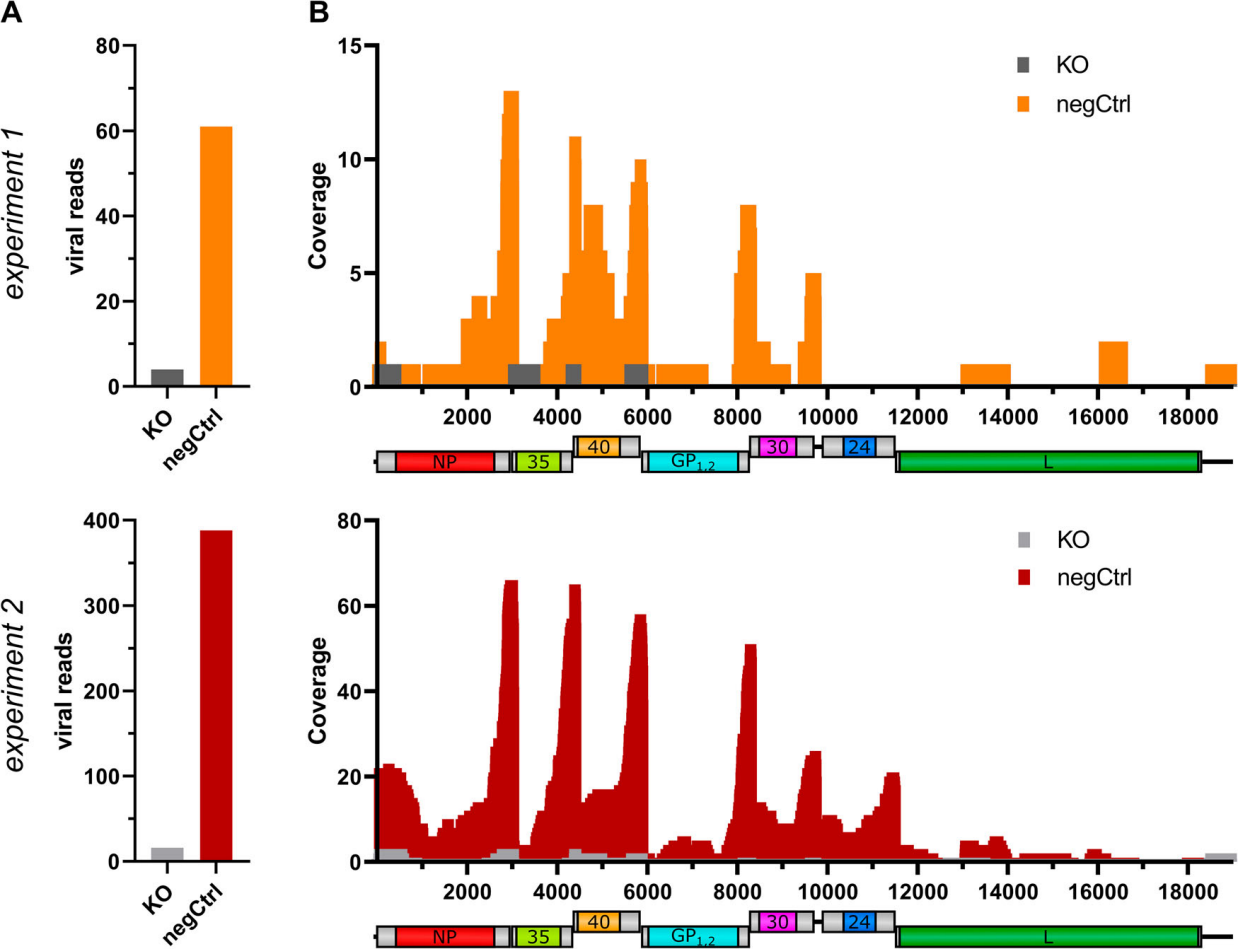
m^6^A methylation of EBOV mRNAs. NegCtrl or METTL3 KO cells were infected with rgEBOV (MOI = 1). 24 hpi, cells were treated with Actinomycin D and 48 hpi, RNA was isolated from infected cells. mRNA was subjected to miCLIP analysis with subsequent MinION sequencing of barcoded samples in one run. Shown are (A) read counts and (B) coverage of the viral genome from two independent experiments.

The main function of METTL3 in the cell is the methylation of cellular mRNAs [2,33,34]. Therefore, we next analysed the m^6^A methylation pattern of both cellular and EBOV mRNA by extracting mRNA from rgEBOV-infected negCtrl and METTL3 KO 293 T cells and subjecting it to miCLIP analysis [26] followed by next generation sequencing (Figure 5). In this assay, crosslinking of an m^6^A-specific antibody with methylated RNA allows specific purification of m^6^A-methylated RNA, but impairs reverse transcription, resulting in either the introduction of mutations within the DRACH motif, or in truncation of the transcript near the methylation site. As expected, we observed a strong reduction in the number of reads mapping to cellular mRNAs in the METTL3 KO cells compared to those in the negCtrl cells, confirming that we had functionally impaired the m^6^A methylation pathway in the KO cells (Suppl. Fig 2C). Further, we readily observed reads that aligned with EBOV sequences in infected negCtrl cells, but hardly any such reads were found in METTL3 KO cells (Figure 5A), demonstrating m^6^A methylation of EBOV mRNA by METTL3. NGS-analysis showed that most of these reads mapped to the 3’ UTRs of EBOV mRNAs (Figure 5B), indicating that it is these regions that are m^6^A methylated. Coverage was highest for the NP and VP35 3’ UTRs and decreased towards the 5’ end of the genome, following the transcriptional gradient of NSVs [35].

### m^6^A machinery is also important for RNA synthesis of other negative-sense RNA viruses

**Figure 6.**
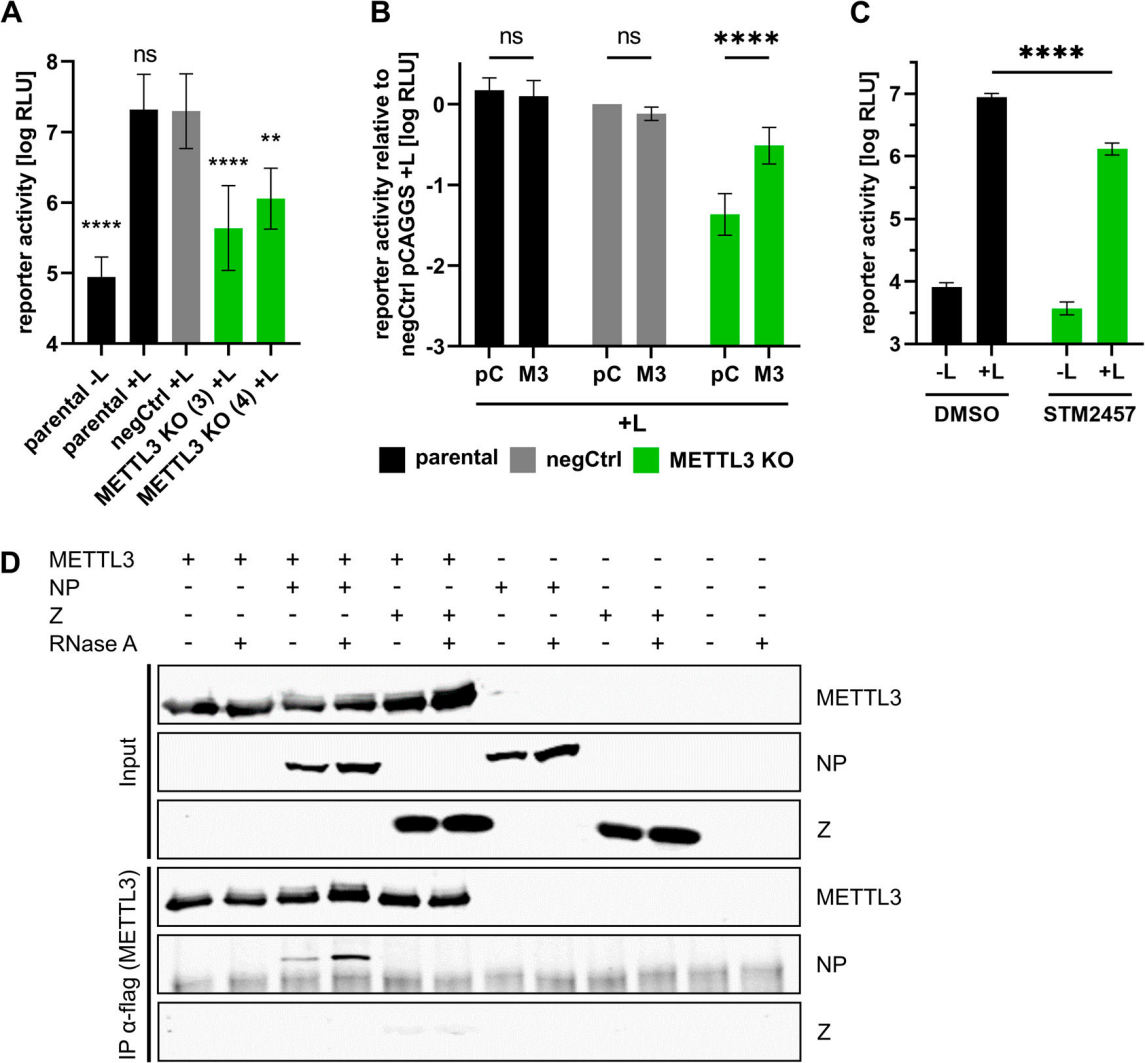
METTL3 is necessary for JUNV RNA synthesis and interacts with JUNV NP. (A, B) Importance of METTL3 for JUNV RNA synthesis and protein expression. Parental, negCtrl or METTL3 KO cell lines were transfected with the plasmids required for a JUNV minigenome assay and in (B) additionally with either empty vector (pC) or pCAGGS-METTL3-ΔgRNA (M3). As a negative control, the viral polymerase was omitted (-L). Means and standard deviations from at least three independent experiments are shown. (C) Influence of METTL3 inhibition on JUNV RNA synthesis and protein expression. 293 T parental cells were transfected with all components for a JUNV minigenome assay. 4 and 24 hpt cells were treated with 30 µM STM2457 or DMSO as control. 48 hpt reporter activity was determined. Means and standard deviations from four biological replicates from two independent experiments are shown. (D) JUNV NP interacts with METTL3. 293 T cells were co-transfected with flagHA-METTL3 and JUNV myc-NP or JUNV myc-Z. Two dpt, cells were lysed and subjected to anti-flag immunoprecipitation. Input and precipitates were subjected to SDS-PAGE and Western blot analysis and METTL3 was detected using anti-HA antibodies and the JUNV proteins using anti-myc antibodies. Representative results from three independent experiments are shown. Asterisks indicate *p* values from one-way ANOVA with Sidak’s multiple comparisons test (**: *p* ≤ 0.01; ****: *p* ≤ 0.0001; ns: *p* ≥ 0.05).

To assess whether the role of m^6^A and METTL3 is specific for the filovirus EBOV or a more common feature of haemorrhagic fever-causing NSVs, we also assessed the importance of METTL3 for the arenavirus JUNV and the orthonairovirus CCHFV. To this end, we performed JUNV minigenome assays in METTL3 KO cells, which resulted in reduced reporter activity compared to parental and negCtrl 293 T cells (Figure 6A). These results indicate that METTL3 is also important for JUNV RNA synthesis and/or protein expression. Complementation of the METTL3 KO with METTL3-ΔgRNA restored reporter activity (Figure 6B), showing the specificity of the observed effect. Further, inhibition of METTL3 with STM2457 led to a similar reduction in reporter activity as METTL3 KO, further confirming the effect observed in the METTL3 KO cell lines (Figure 6C). Analysis of a possible interaction between selected JUNV proteins and METTL3 showed that, like EBOV NP, JUNV NP also interacts with METTL3 in an RNA-independent manner (Figure 6D).

**Figure 7.**
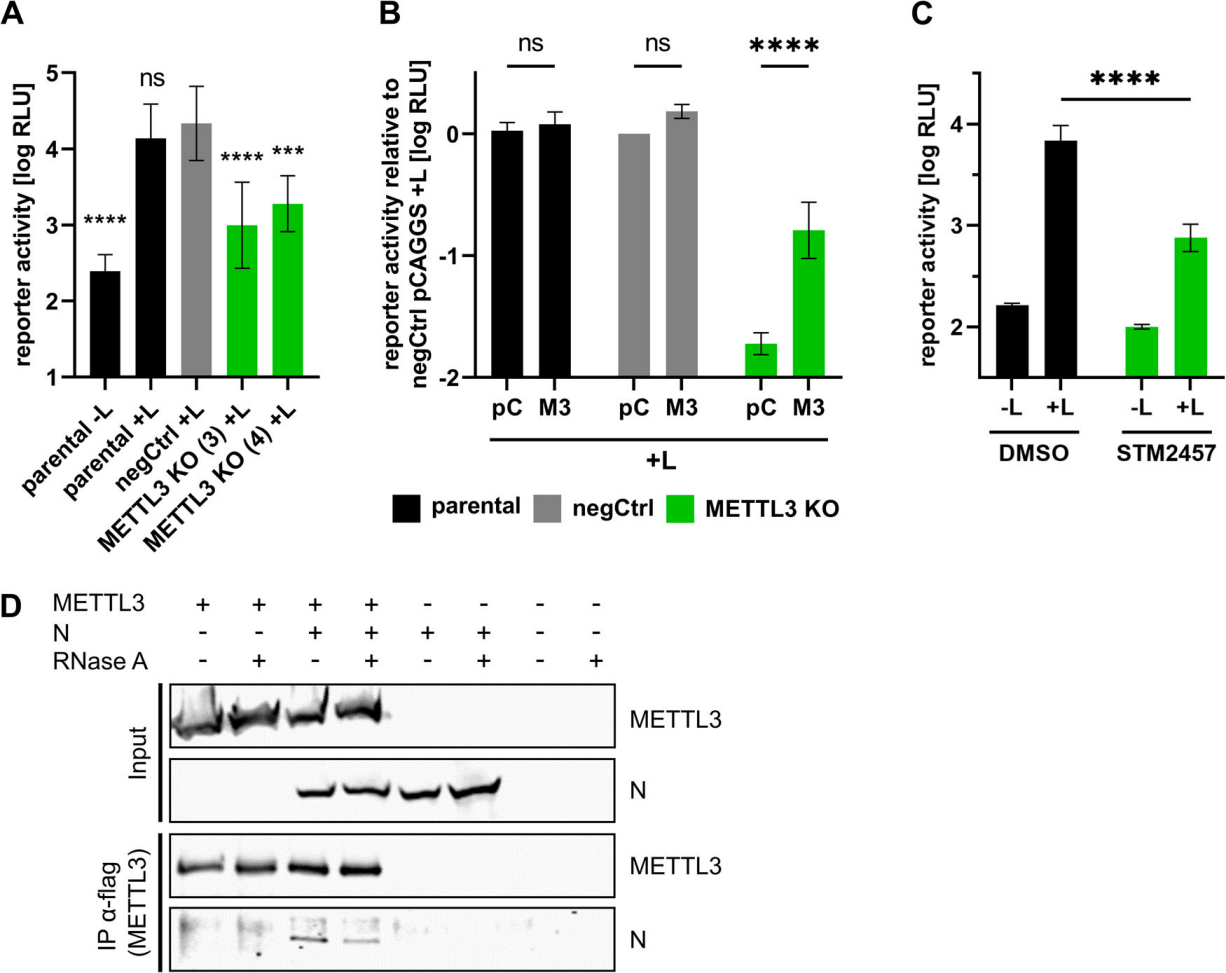
METTL3 is necessary for CCHFV RNA synthesis and interacts with CCHFV N. (A, B) Importance of METTL3 for CCHFV RNA synthesis and protein expression. Parental, negCtrl or METTL3 KO cell lines were transfected with the components for a CCHFV minigenome assay and in (B) additionally with either empty vector (pC) or pCAGGS-METTL3-ΔgRNA (M3). As a negative control, the viral polymerase was omitted (-L). Means and standard deviations from at least three independent experiments are shown. (C) Influence of METTL3 inhibition on CCHFV RNA synthesis and protein expression. 293 T parental cells were transfected with all components for a CCHFV minigenome assay. 4 and 24 hpt cells were treated with 30 µM STM2457 or DMSO as control. Reporter activity was determined 48 hpt. Means and standard deviations from four biologicals replicates from two independent experiments are shown. (D) METTL3 interacts with CCHFV N. 293 T cells were co-transfected with flagHA-METTL3 and CCHFV N. Two dpt, cells were lysed and subjected to anti-flag immunoprecipitation. Input and precipitates were analysed via SDS-PAGE and Western blot analysis and METTL3 was detected using anti-HA antibodies and CCHFV N with protein-specific antibodies. Representative results from three independent experiments are shown. Asterisks indicate *p* values from one-way ANOVA with Sidak’s multiple comparisons test (***: *p* ≤ 0.001; ****: *p* ≤ 0.0001; ns: *p* ≥ 0.05).

Similarly, CCHFV minigenome assays in METTL3 KO cells showed a strong reduction in reporter activity (Figure 7A), demonstrating that METTL3 is important for CCHFV RNA synthesis and/or protein expression as well. Complementation of the METTL3 KO also increased reporter activity, confirming the specificity of the effect (Figure 7B). Additionally, inhibition of METTL3 by STM2457 led to a strong reduction in reporter activity, resembling the effect observed upon METTL3 KO and thereby confirming the effect of the METTL3 KO (Figure 7C). Further, immunoprecipitations of flagHA-METTL3 led to the coprecipitation of the CCHFV nucleoprotein (N) in the presence and absence of RNase A, showing that also CCHFV N interacts with METTL3 in an RNA-independent manner (Figure 7D).

### Loss of METTL3 does not lead to elevated interferon induction in response to EBOV, JUNV or CCHFV RNA synthesis

**Figure 8.**
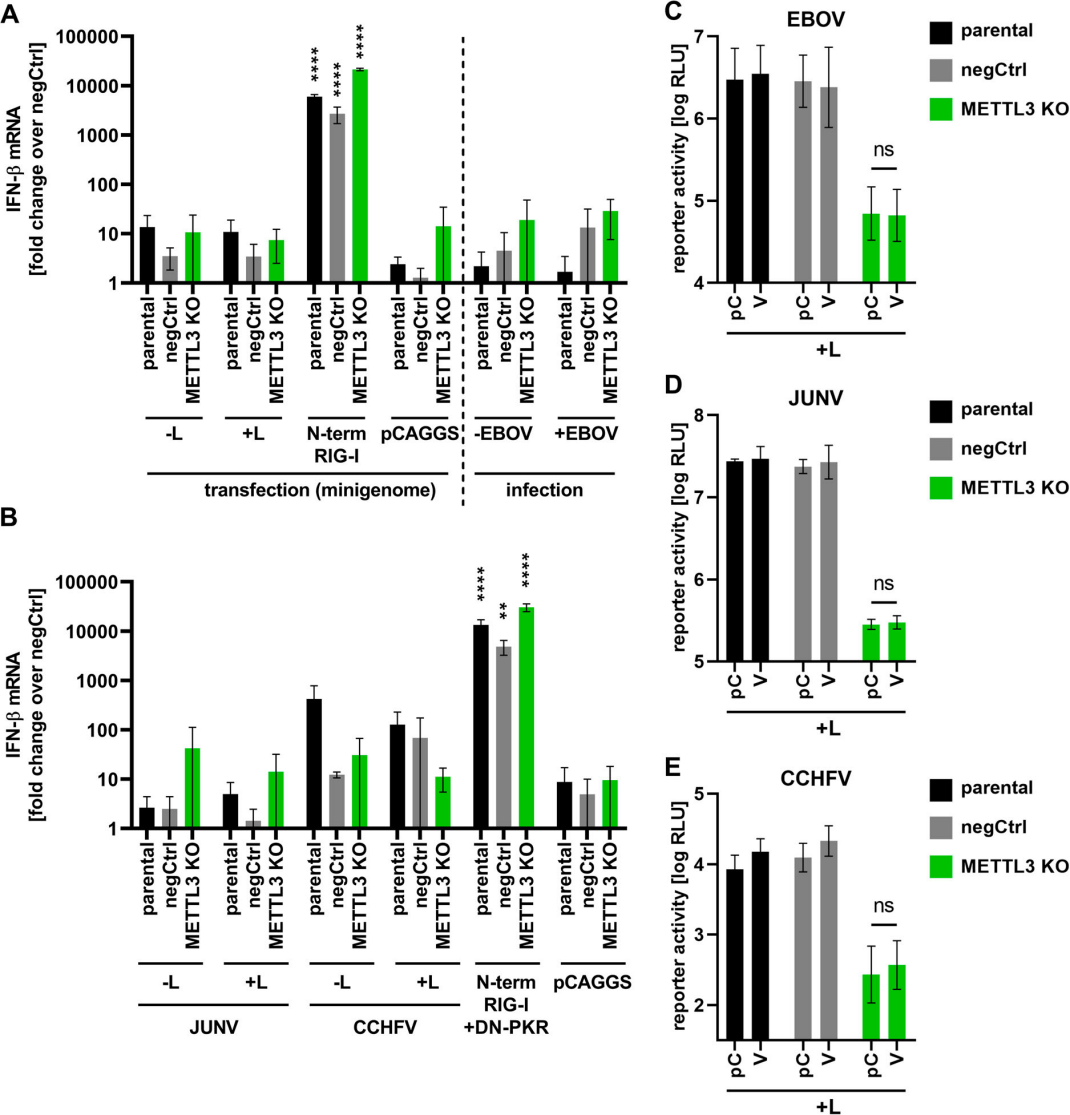
METTL3 KO has no influence on the cellular IFN response. (A) Parental, negative control (negCtrl) or METTL3 KO cells were transfected with the components for an EBOV minigenome, CARD domains of RIG-I (N-term RIG-I; positive control) or infected with rgEBOV (MOI = 0.5). RNA was isolated two dpt or 24 hpi and subjected to RT-qPCR for IFN-β and GAPDH mRNAs. (B) Parental, negCtrl or METTL3 KO cells were transfected with the components for a JUNV minigenome, a CCHFV minigenome or N-term RIG-I (positive control). All minigenome samples, as well as the positive controls, were additionally transfected with dominant negative protein kinase R (DN-PKR). Two dpt, RNA was isolated and analysed via RT-qPCR for GAPDH and IFN-β levels. All cell lines were transfected with the components for (C) EBOV, (D) JUNV or (E) CCHFV minigenome assays as well as either empty vector (pC) or PIV5 V (V). As a negative control, the polymerase was omitted. Two dpt, reporter activities were measured. Means and standard deviations from at least two independent experiments are shown. Asterisks indicate *p* values from one-way ANOVA with Dunett’s (B) or Sidak’s (A, C, D, E) multiple comparison’s test (**: *p* ≤ 0.01; ****: *p* ≤ 0.0001; ns: *p* ≥ 0.05). Statistics in panels (A) and (B) are calculated in relation to pCAGGS-transfected negCtrl cells.

Previous studies have shown that, for some NSVs, the loss of m^6^A results in an elevated IFN-β response in infected cells [15,16,36]. To assess whether the methylation of EBOV RNAs serves the same purpose, we performed minigenome assays and infections in parental, negCtrl and METTL3 KO cells and determined the levels of IFN-β mRNA in these cells (Figure 8A). As a positive control, we transfected the CARD domains of RIG-I [37], which resulted in robust IFN-β mRNA levels in all three cell lines. However, neither EBOV minigenome assays nor EBOV infections resulted in a significant increase in IFN-β induction in METTL3 KO cells, and similar results were also obtained for JUNV and CCHFV minigenome assays (Figure 8B).

To further exclude a role of IFN-β, we also performed EBOV minigenome assays in the presence and absence of parainfluenza virus 5 (PIV5) V, which is known to be a strong inhibitor of both IFN-β induction and signalling [38–40] (Figure 8C; Suppl. Fig. 3A-B). However, PIV5 V did not increase reporter activity in METTL3 KO cells, further demonstrating that the reduction in viral RNA synthesis upon METTL3 KO is not due to an elevated IFN-β response. Again, similar results were obtained for JUNV (Figure 8D) and CCHFV (Figure 8E), supporting the observation that the main function of METTL3 in the life cycles of haemorrhagic fever-causing NSVs is not due to inhibition of the IFN-β response.

## Discussion

It has been described for many viruses that m^6^A influences their viral life cycle, albeit with very different outcomes. Antiviral roles have been shown for some viruses, such as flaviviruses or Kaposi sarcoma virus [13,41], but proviral roles are reported for others [15,16,36]. Here, we have shown that METTL3 interacts with EBOV NP and VP30, as well as JUNV NP and CCHFV N, that it gets recruited into viral inclusion bodies, and that it methylates EBOV mRNA. Further, METTL3 KO as well as inhibition of METTL3 resulted in a clear reduction of RNA synthesis in EBOV, JUNV and CCHFV minigenome assays, results that were confirmed in EBOV infection.

Recently, generation of METTL3 KO cell lines has been shown to result in expression of alternative METTL3 versions from alternative spliceoforms [42]. While we cannot exclude that our 293 T METTL3 KO cell lines generate these alternative spliceoforms in order to compensate the KO, our results show that we have only low levels of residual m^6^A in cellular mRNAs in our KO cell lines. Furthermore, we only used low passages of our KO cell clones, as extensive passaging increases the chances for a functional complementation. Also, the METTL3 KO clones showed reduced growth in comparison to parental and negCtrl cell lines, suggesting that METTL3 is absent in these low passages. Finally, inhibition of METTL3 with STM2457 led to the same results as METTL3 KO, confirming the results obtained in the KO cell lines.

The phenotypes observed in our study show parallels to those observed upon methyltransferase depletion or deletion in other NSV infections [15,16,36]. However, in the case of the NSVs VSV and HMPV, m^6^A methylation of RNAs has been described to inhibit the cellular immune response, and specifically IFN-β induction, either by masking viral RNAs so that they resemble host RNAs or by preventing the formation of dsRNAs that would trigger activation of RIG-I and MDA5 [15,16]. Our results show that there is no significant increase in IFN-β induction in METTL3 KO cells upon infection with EBOV, and that the same was true in the context of JUNV or CCHFV minigenome assays, demonstrating that m^6^A methylation of viral RNAs has a different function in the life cycles of EBOV, JUNV and CCHFV than has been described for other NSVs. Rather, other known functions of m^6^A methylation are more likely responsible for the negative effect of METTL3 KO on the life cycles of the analysed viruses, such as its impact on RNA stability, translational efficiency and/or mRNA export [7,9,43–45].

In particular, m^6^A methylation of cellular mRNAs can promote their nuclear export via the nuclear RNA export factor 1 (NXF1) pathway through interaction of the m^6^A reader YTHDC1 with the export adapter SRSF3 [7]. Also, hepatitis B virus uses YTHDC1 for export of m^6^A methylated viral RNAs from the nucleus [46]. We could previously demonstrate that EBOV mRNAs are exported from viral inclusion bodies by NXF1 [24,47], and MS data suggest an interaction of EBOV VP30 with YTHDC1 [17]. Indeed, impaired RNA export from inclusion bodies could explain the observed reduction in RNA synthesis and protein expression. However, while JUNV also replicates in cytoplasmic inclusion bodies and requires NXF1, NXF1 is dispensable for CCHFV RNA synthesis and protein expression, which makes it an unlikely explanation for the observed phenotype in the CCHFV minigenome assays [47,48].

Furthermore, binding of m^6^A readers like YTHDF to m^6^A mRNA has been shown to cause liquid–liquid phase separation of YTHDF-mRNA complexes [49]. As inclusion bodies of many NSVs have been described to be liquid organelles, and also EBOV inclusion bodies show some characteristics of liquid organelles [30,50,51], the interaction between YTHDF proteins and EBOV m^6^A mRNA might support the phase separation process or help to separate mRNAs from other RNA-binding proteins like NP, which might also explain the localization of METTL3 in punctae within the inclusion bodies.

Reduction of mRNA stability was one of the first described functions of m^6^A [52], but accelerated degradation of viral mRNAs is unlikely to lead to more efficient viral RNA synthesis. However, enhanced translation of viral mRNAs could explain the observed phenotype, especially since m^6^A has been suggested to enhance translation through different methods, all involving recruitment of eukaryotic initiation factor 3 (eIF3), which has also been shown to interact with EBOV VP35, although the functional consequences of this interaction are not well understood [43–45,53].

Taken together, we have shown that METTL3 methylates EBOV mRNAs, is recruited to EBOV inclusion bodies as the sites of viral RNA synthesis, and plays an important role in viral RNA synthesis of not only EBOV but also other haemorrhagic fever-causing NSVs (i.e. JUNV and CCHFV). Further, we show that a METTL3 inhibitor, which has been shown to be effective against acute myeloid leukaemia and in an improved version is currently undergoing clinical trials for solid tumours (identifier NCT05584111) [32], leads to inhibition of all three viruses. Similarly, inhibitors of the S-adenosylhomocysteine hydrolase, which is required for the synthesis of S-adenenosylmethionin (SAM), the methyl-donor for m^6^A, has been shown to protect mice from an otherwise lethal EBOV infection [54–56]. However, SAM is also the substrate for cap-methylation and the effects observed in these studies have been suggested to be due to the loss of the cap, although it could be due to both loss of the cap-methylation and m^6^A. In order to identify the exact function of m^6^A in the life cycles of these viruses, further studies assessing the importance of other components of the m^6^A writer complex like RBM15 and WTAP, which we identified as important host factors for EBOV RNA synthesis in a genome-wide siRNA screen [20], but also the role of the individual m^6^A reader proteins will be required. Nevertheless, the broad range of viruses for which m^6^A methylation seems to play an important role in facilitating their life cycle suggests that the m^6^A machinery is a promising target for broadly-acting antiviral drugs.

## Supplementary Material

Supplemental Material

Supplemental Material

## Data Availability

The sequencing data from miCLIP analysis are available under 10.6084/m9.figshare.21716981, and the authors confirm that all other data supporting the findings of this study are available within the article and its supplementary material.
