# Supplementary material for: N6-methyladenosine is required for efficient RNA synthesis of Ebola virus and other haemorrhagic fever viruses
Source: Emerg Microbes Infect. 2023 Jun 21;12(2):2223732. doi: 10.1080/22221751.2023.2223732 (PMC10286672; doi:10.1080/22221751.2023.2223732)
Supplement: Supplemental Material [file TEMI_A_2223732_SM1863.pdf]

Supplemental Figures

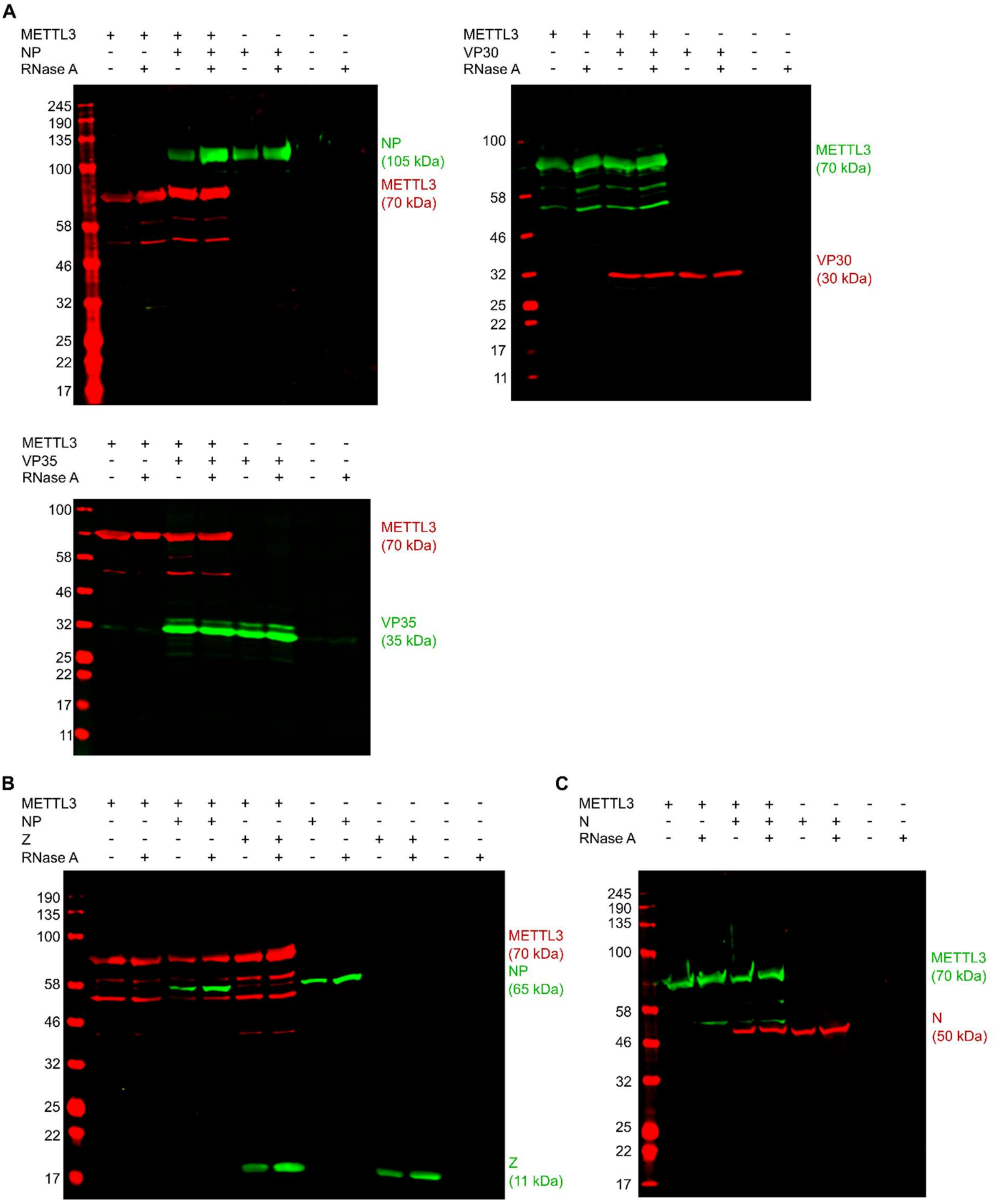

Suppl. Figure 1. Western Blot raw data. Unmodified versions of the Input Western Blots shown in Figures 1, 6C and 7C. Indicated are the molecular weights for the protein marker used as well as the expected molecular weights (kDa) for the proteins detected on each blot: (A) EBOV NP, VP30, VP35, (B) JUNV NP and Z, (C) CCHFV N.

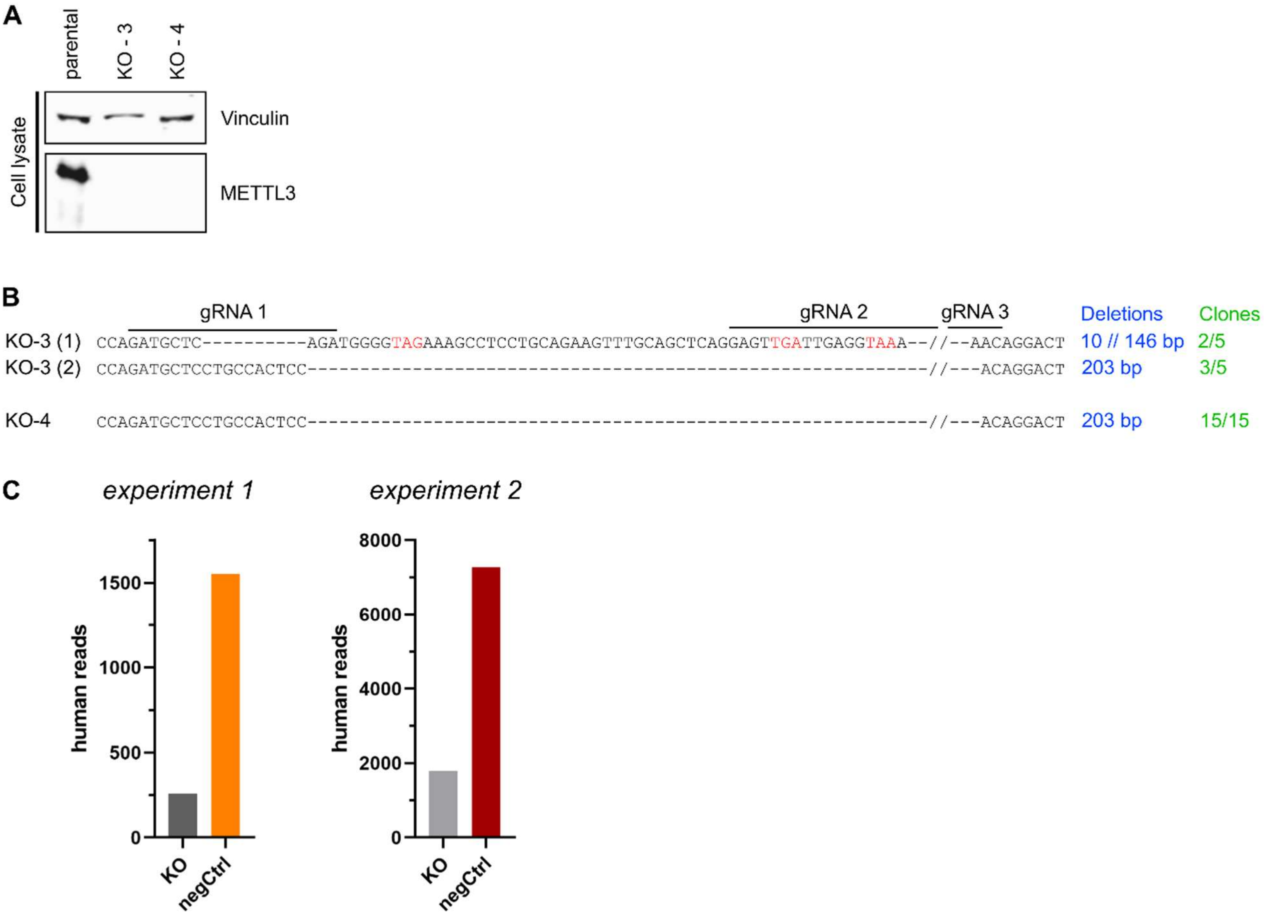

Suppl. Figure 2. Characterization of 293T METTL3 KO cell lines. (A) METTL3 expression in KO cell lines. Parental and METTL3 KO cells were harvested and analysed via SDS-PAGE and Western blot using antibodies against METTL3 and vinculin (as a control). (B) Sequencing of METTL3 KO cell lines. Genomic DNA was isolated from METTL3 KO cell lines and region of the *METTL3* gene targeted by the gRNAs used for KO generation was amplified via PCR. Purified PCR products were ligated into an expression vector and transformed into competent E. coli. 5 and 15 clones were sent for sequencing, resulting in two sequences for clone 3 and one sequence for clone 4. (C) Analysis of miCLIP reads for human reads. NegCtrl or METTL3 KO cells were infected with rgEBOV (MOI = 1). 24 hpi, cells were treated with Actinomycin D and 48 hpi, RNA was isolated from infected cells. mRNA was subjected to miCLIP analysis with subsequent MinION sequencing of barcoded samples in one run. Reads were reanalysed by Fastq Human Alignment (GRCh38) to identify human mRNA reads. Shown are read counts for both experiments.

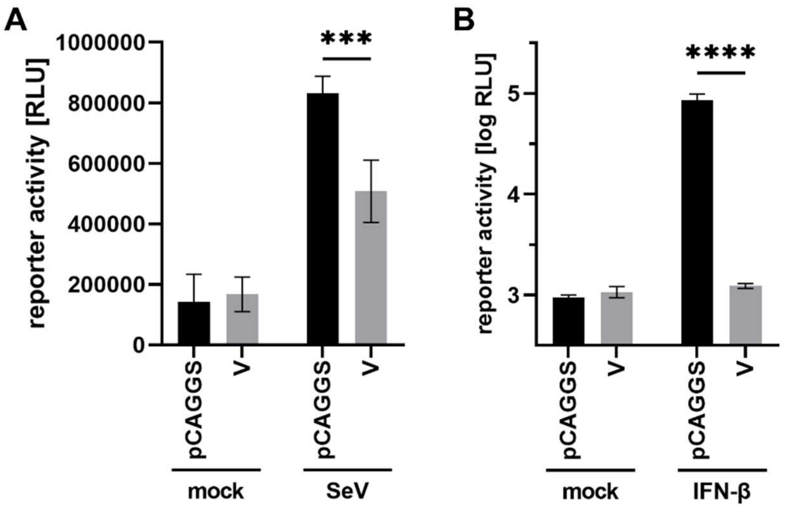

Suppl. Figure 3. Control experiments for analysis of IFN-β response. (A) PIV5 V blocks IFN-β induction in 293T cells. Parental 293T cells were transfected with a vector expressing Firefly luciferase under control of an IFN-β promotor as well as pCAGGS-PIV5-V or empty vector. 24 hpt cells were infected with Sendai virus (SeV) at an MOI of 200 or mocj infected. Another 24 h later, reporter activity was determined. (B) PIV5 V block IFN-β signalling in 293T cells. Parental cells were transfected with a vector expressing Firefly luciferase under control of an interferon-sensitive response element (ISRE) as well as pCAGGS-PIV5-V or empty vector. 24 hpt cells were stimulated with 62.5 pg IFN-β or remained unstimulated. 8 h after stimulation, reporter activity was determined. Means and standard deviations from two (B) or four (A) biological replicates from two independent experiments are shown. Asterisks indicate p values from one-way ANOVA with Sidak's multiple comparison's test (***: $p \leq 0.001$; ****: $p \leq 0.0001$; ns: $p \geq 0.05$).
