# Supplementary material for: N6-methyladenosine is required for efficient RNA synthesis of Ebola virus and other haemorrhagic fever viruses
Source: Emerg Microbes Infect. 2023 Jun 21;12(2):2223732. doi: 10.1080/22221751.2023.2223732 (PMC10286672; doi:10.1080/22221751.2023.2223732)
Supplement: Supplemental Material [file TEMI_A_2223732_SM1858.pdf]

Supplemental methods

Plasmids, antibodies and chemicals

Expression plasmids for the EBOV RNP proteins, the T7 polymerase, as well as the T7-driven monocistronic minigenome and the PolII-driven replication-deficient minigenomes (pT7-1cis-vRNA-hrluc, pCAGGS-1cis-vRNA-nluc-RdM) used in this study have been described previously [1-3]. The plasmids for the expression of untagged or N-terminally flag/HA-tagged METTL3 were generated by inserting the *METTL3* gene into a pCAGGS or pCAGGS-flag/HA-vector, respectively. For the generation of pCAGGS-METTL3-ΔgRNA, silent mutations were introduced into the binding sites for the four guide RNAs (gRNAs) by exchanging every third nucleotide corresponding to the next most frequently used codon for each amino acid. The plasmids for the expression of constitutively active RIG-I (pEF-RigI-Nterm), dominant-negative PKR (pI.18-HA-PKRdelE7) as well as parainfluenza virus 5 V (pCAGGS-PIV5-V) have been previously described [4-6]. Also, the plasmid encoding firefly luciferase under control of an IFN-beta promotor (pI125-luc), the plasmid encoding firefly luciferase under control of an interferon-stimulated response element (ISRE; pISRE-Luc) and the expression plasmid for Renilla luciferase have been previously described [7].

The expression plasmids for the JUNV RNP proteins, the T7-driven JUNV minigenome (pAMP-JUNV-S-mg-nluc-1L/2L), the CCHFV RNP proteins and the T7-driven CCHFV minigenome (pT7ribo-10200-L-HHR-rluc) have been described previously [5,8]. The expression plasmids for myc-tagged JUNV proteins were generated by subcloning the respective open reading frame into a pCAGGS-myc vector. Detailed cloning strategies are available on request.

For generation of clonal knockout (KO) cell lines, four different gRNA sequences were inserted into the plasmid pX330-eCas9-PuroR (encoding modified Cas9 and a puromycin resistance gene for selection) to allow METTL3 KO cell line generation. The pX330-eCas9-

PuroR plasmid is based on pX330-U6-Chimeric_BB-CBh-hSpCas9 (Addgene #42230 [9]). After insertion of the neomycin resistance (neoR) expression cassette from pcDNA3.1(+) (Thermo Fisher Scientific) as described previously [10], the neoR ORF was replaced by a PCR amplified puromycin resistance (puroR) ORF derived from the lentivirus vector pGIPZ (Open Biosystems, kindly provided by Stefan Finke, FLI). Finally, three amino acid substitutions were introduced into the Cas9 nuclease by site directed mutagenesis of the plasmid, resulting in expression of eSpCas9(1.1) which is considered to possess enhanced specificity [11].

For Western Blot analyses, anti-flag (Sigma-Aldrich, cat. no. F1804; 1:2000), anti-myc (Thermo Fisher Scientific, cat. no. PA1-981; 1:1000), anti-METTL3 (Thermo Fisher Scientific, cat. no. MA5-35248; 1:1000), anti-HA (Abcam, cat. no. ab9111; 1:2000) and anti-Vinculin (Santa Cruz, cat. no. sc73614; 1:1000) antibodies were used as well as fluorescently labelled anti-mouse (Alexa Fluor 680, Jackson Immunoresearch, cat. no. 115-625-174; 1:40.000), anti-chicken (Alexa Fluor 680, Jackson Immunoresearch, cat. no. 703-625-155; 1:50.000) and anti-rabbit (Alexa Fluor 790, Jackson Immunoresearch, cat. no. 111-655-144; 1:50.000) antibodies. For detection of CCHFV N, a mouse monoclonal antibody (clone 3E4B1; 1:50) was produced by the FLI biobank as previously described [12] and following approved protocols using recombinantly expressed N protein (Strain IbAr 10200) as the immunogen.

For immunofluorescence analyses, anti-HA (Abcam, cat. no. ab9111; 1:1000), anti-METTL3 (Thermo Fisher Scientific, cat. no. MA5-35248; 1:200) and previously described in-house generated anti-EBOV VP30 [13] (1:300) antibodies were used together with fluorescently labelled secondary antibodies against mouse (Alexa Fluor 568, Thermo Fisher Scientific, cat. no. A-11031; 1:500), rabbit (Alexa Fluor 488, Abcam, cat. no. ab150077; 1:1200) and chicken (Alexa Fluor 647, Thermo Fisher Scientific, cat. no. A-21449; 1:1200). A m^6A-specific antibody (abcam, cat. no. ab151230) was used for miCLIP and anti-flag antibody (Sigma-Aldrich, cat. no. F1804; 1 µl/10 µl beads) was used for coIP analyses.

The METTL3 inhibitor STM2457 was purchased from MedChemExpress and a 10 mM stock solution was prepared with DMSO. IFN-beta was obtained from R&D systems and a stock solution was prepared following the manufacturer's instructions.

Co-immunoprecipitation of viral proteins

Two days after transfection, 293T cells were harvested and lysed for 2 h using a 1 % NP-40 buffer (1% NP-40; 50 mM Tris; 150 mM NaCl; pH 7.4) by rotating at 4 °C. The pre-immune lysate was cleared by centrifugation and 750 ul were used for immunoprecipitation, while 150 ul (representing 20 % of the IP sample) were subjected to acetone precipitation (input control). CoIP was performed with Protein G Dynabeads (Thermo Fisher Scientific) coupled to anti-flag antibodies. Immunoprecipitation was performed for 10 min at RT and samples were subsequently analysed via SDS-PAGE and Western Blot.

Generation of METTL3 KO cell line

For the generation of a HEK 293T KO cell line, cells were transfected with four different Cas9 and *METTL3*-specific gRNA-expressing plasmids (500 ng each; pX330-eCas9-PuroR) or a plasmid expressing the negative control gRNA and selected using 3 ng/ul puromycin (Thermo Fisher Scientific) for two weeks. For gRNAs targeting exon 3 of *METTL3* the following sequences were used: 5'-GATGCTCCTGCCACTCAAGA-3', 5'-GAGTTGATTGAGGTAAAGCG-3', 5'- GCAGAAGCGGCGTGCAGAAC-3', 5'-TGCTGCCTCAGATGTTGATC-3'. For generation of the negCtrl cell line the following gRNA was used: 5'-GTTCCGCGTTACATAACTTA-3' Single cell clones were transferred to 12-well plates and KO was validated via Western Blot analyses.

For the genomic characterization of the KO cell lines, genomic DNA was isolated using QIAamp DNA Mini Kit (Qiagen). Parts of the *METTL3* gene including exon 3 were amplified via PCR (METTL3-fwd: 5'-TAT AGG TAA CAC TGT TGG CCC C-3'; METTL3-rev: 5'-GTT GAC ACT TTG GGA CAG ACA A-3'), purified and ligated into pJET1.2 using the

CloneJET kit (Thermo Fisher Scientific) according to the manufacturer's instructions. The resulting plasmid was transformed into competent *E. coli* and 5-15 bacterial clones were analysed via Sanger sequencing using the same primers as for the PCR.

EBOV minigenome assays

For the classical minigenome assay, HEK 293T cells were transfected with the plasmids encoding the RNP proteins (NP, VP35, VP30 and L), which are necessary and sufficient to allow viral RNA synthesis, the T7 polymerase, firefly luciferase (as a transfection control), and the minigenome plasmid. This minigenome plasmid encodes a miniature version of the viral genome, in which all viral genes have been removed and replaced by a luciferase (nanoluc or Renilla luciferase) reporter gene, but which still contains the non-coding terminal genome regions containing the viral promoter sequences required to be recognized as an authentic template for viral RNA synthesis (replication and transcription) by the RNP proteins, under control of a T7 promoter for initial minigenome RNA transcription. For the replication-deficient minigenome, instead of the classical minigenome plasmid, a plasmid encoding a version of the minigenome that lacked 55 nt of the antigenomic replication promoter was transfected. This results in a minigenome that can still undergo transcription, but can no longer be replicated by the viral polymerase. This renders transcription, which in a classical minigenome assay is influenced by replication (due to the number of genome templates available for transcription), independent of genome replication.

For all assays, cells were lysed two days post-transfection with 1x Lysis Juice (PJK) for 10 min and lysates were cleared by centrifugation at 10,000 x g for 3 min. 40 µl of the cleared lysate was combined with 40 µl luciferase substrate (beetle juice [PJK], Renilla Glow Juice [PJK] or nanoGlo [Promega]) in black 96-well plates. Luciferase activity was measured using a GloMax Discover (Promega) plate reader, and reporter activities (Renilla or nanoluc activity) were normalised to control luciferase (firefly) activities.

For the complementation assay, minigenome assays were performed as described above, but cells were additionally transfected with empty vector (pCAGGS) or pCAGGS-METTL3-ΔgRNA, which contains silent mutations in all gRNA-binding sites. For analysing the influence of METTL3 KO on IFN-β induction, cells were additionally transfected with either empty vector or pCAGGS-PIV5-V and reporter activity was determined as described above.

For the inhibitor assay, minigenome assays were performed as described above, but 4 hpt and 24 hpt, cell culture supernatant was replaced by media containing either 30 µM STM2457 (in DMSO) or the corresponding amount of DMSO. Reporter activity was determinded 48 hpt as described above.

miCLIP

For miCLIP analysis, mRNA was diluted in IP buffer (50 mM Tris, pH7.4; 100 mM NaCl; 0.05 % NP-40) to 450 µl and incubated with 5 µg m^6A antibody for 2 h while rotating at 4 °C. Solutions were transferred to 6-well plates and UV crosslinking (254 nm) was performed twice with 0.15 J/cm^2. Crosslinked antibody-RNA mix was transferred into reaction tubes containing 50 µl Protein G coupled Dynabeads (Thermo Fisher Scientific) and incubated for 1 h while rotating at 4 °C. Afterwards, beads were washed twice with High Salt buffer (50 mM Tris, pH 7.5; 1 M NaCl, 1 mM EDTA, 1 % NP-40; 0.1 % SDS), twice with IP buffer and twice with polynucleotide kinase wash buffer (20 mM Tris, 10 mM $MgCl_2$, 0.2 % Tween-20). On-bead proteinase K digest was performed by resuspending the beads in proteinase K buffer (0.1 M NaCL, 10 mM Tris pH 8.0, 1 mM EDTA, 0.5 % SDS, 200 µg/ml Proteinase K) and incubating at 50 °C for 1 h. RNA was isolated using TrizolLS according to the manufacturer's instruction.

MinION Sequencing

For preparation of the library with the Direct cDNA Sequencing with Native Barcoding Kit (Oxford Nanopore, cat. no. SQK-DCS109 and EXP-NBD104) all mRNA samples were barcoded and pooled into one library, following the manufacturer's instructions. The library

was sequenced using Flongle flow cells in a MinION Mk1 (Oxford Nanopore) device. Basecalling was done using the MinKNOW software and reads were analysed using the EPI2ME Fastq Custom Alignment workflow with the EBOV genome sequence (Genbank accession NC 002549.1) as reference for alignments with the viral genome. For alignment with the human genome, the EPI2ME Fastq Human Alignment GRCh38 was used.

Minigenome assays of other NSVs

For JUNV and CCHFV minigenome assays, parental, negCtrl and METTL3 KO cells were seeded in 12-well plates and one day later transfected with the plasmids encoding T7-polymerase, dominant-negative PKR, firefly luciferase (as a control), and the respective minigenome plasmids. For the JUNV minigenome systems additionally pCAGGS-JUNV-NP and pCAGGS-JUNV-L were cotransfected, while for the CCHFV minigenome, cells were additionally transfected with the plasmids encoding CCHFV N and L. As a negative control, the plasmid encoding the respective viral polymerase was omitted. Two days post transfection, cells were lysed with 1x Lysis Juice (PJK) for 10 min before cell lysates were cleared by centrifugation at 10,000 xg. For measurement of luciferase activity, 40 µl cleared cell lysate was combined with 40 µl substrate (beetle juice, NanoGlo or Renilla Glow Juice) in black 96-well plates and analysed using a GloMax Discover (Promega) plate reader.

As described above for the EBOV minigenome, for complementation assays, cells were additionally transfected with empty vector (pCAGGS) or pCAGGS-METTL3-ΔgRNA and for determining the impact of METTL3-KO on IFN-beta induction, cells were additionally transfected with either empty vector or pCAGGS-PIV5-V. Otherwise, minigenome assays were performed as described above. Inhibitor assays have been performed as described above for the EBOV minigenome assay.

PIV5 V Testing

For the analysis of the inhibitory function of PIV5 V on IFN-β induction, 293T cells in 12-well plates were transfected with an expression plasmid encoding firefly luciferase under control of the IFN-β-promotor (pI125-luc) as well as either empty vector or PIV5 V expression vector and a Renilla luciferase expression vector as control. 24 hpt, cells were infected with Sendai virus at an MOI of 200 or mock infected, and another 24 h later cells were harvested. To this end, cells were lysed with 200 µl Glo Lysis Buffer for 10 min at room temperature and lysates were cleared by centrifugation. For measurement of reporter activity, 40 µl of pre-cleared lysate was added to 40 µl Bright-Glo or 40 µl Renilla Glow Juice in black opaque 96-well plates and measured in a GloMax Discover plate reader. Firefly luciferase activity was normalised to Renilla luciferase activity.

For analysis of the inhibition of IFN-β signalling by PIV5 V, 293T cells in 12-well plates were transfected with an expression plasmid encoding firefly luciferase under control of an ISRE (pISRE-Luc) as well as either empty vector or PIV5 V expression vector and a Renilla luciferase expression vector as control. 24 hpt cells were either stimulated by addition of 62.5 pg IFN-β or remained unstimulated, and 8 h later reporter activity was measured. To this end, cells were lysed in 200 µl H20 with 1 % Triton X-100 for 10 min at room temperature and lysates were cleared by centrifugation. 40 µl pre-cleared lysate was added to 40 µl Renilla Glow Juice or 40 µl Beetle Juice in black opaque 96-well plates and reporter activity was measured in a GloMax Discover plate reader. Firefly luciferase activity was normalised to Renilla luciferase activity.

Statistical analyses

One-way ANOVA with Dunett's multiple comparisons test (comparisons to a control; Fig. 8B) or Sidak's multiple comparisons test (selected comparisons; Fig. 3, 4, 6, 7, 8A, 8C-D) was performed using the GraphPad Prism 8 software.

References:

1. Brandt J, Wendt L, Bodmer BS, et al. The Cellular Protein CAD is Recruited into Ebola Virus Inclusion Bodies by the Nucleoprotein NP to Facilitate Genome Replication and Transcription. Cells. 2020 May 1;9(5).

2. Hoenen T, Jung S, Herwig A, et al. Both matrix proteins of Ebola virus contribute to the regulation of viral genome replication and transcription. Virology. 2010 Jul 20;403(1):56-66.

3. Martin S, Chiramel AI, Schmidt ML, et al. A genome-wide siRNA screen identifies a druggable host pathway essential for the Ebola virus life cycle. Genome Med. 2018 Aug 7;10(1):58.

4. Klemm C, Reguera J, Cusack S, et al. Systems to establish bunyavirus genome replication in the absence of transcription. J Virol. 2013 Jul;87(14):8205-12.

5. Pickin MJ, Devignot S, Weber F, et al. Comparison of Crimean-Congo Hemorrhagic Fever Virus and Aigai Virus in Life Cycle Modeling Systems Reveals a Difference in L Protein Activity. J Virol. 2022 Jul 13;96(13):e0059922.

6. Rothenfusser S, Goutagny N, DiPerna G, et al. The RNA helicase Lgp2 inhibits TLR-independent sensing of viral replication by retinoic acid-inducible gene-I. J Immunol. 2005 Oct 15;175(8):5260-8.

7. Dunham EC, Banadyga L, Groseth A, et al. Assessing the contribution of interferon antagonism to the virulence of West African Ebola viruses. Nat Commun. 2015 Aug 5;6:8000.

8. Dunham EC, Leske A, Shifflett K, et al. Lifecycle modelling systems support inosine monophosphate dehydrogenase (IMPDH) as a pro-viral factor and antiviral target for New World arenaviruses. Antiviral Res. 2018 Sep;157:140-150.

9. Cong L, Ran FA, Cox D, et al. Multiplex genome engineering using CRISPR/Cas systems. Science. 2013 Feb 15;339(6121):819-23.

10. Hubner A, Petersen B, Keil GM, et al. Efficient inhibition of African swine fever virus replication by CRISPR/Cas9 targeting of the viral p30 gene (CP204L). Sci Rep. 2018 Jan 23;8(1):1449.

11. Slaymaker IM, Gao L, Zetsche B, et al. Rationally engineered Cas9 nucleases with improved specificity. Science. 2016 Jan 1;351(6268):84-8.

12. Bussmann BM, Reiche S, Jacob LH, et al. Antigenic and cellular localisation analysis of the severe acute respiratory syndrome coronavirus nucleocapsid protein using monoclonal antibodies. Virus Res. 2006 Dec;122(1-2):119-26.

13. Wendt L, Brandt J, Bodmer BS, et al. The Ebola Virus Nucleoprotein Recruits the Nuclear RNA Export Factor NXF1 into Inclusion Bodies to Facilitate Viral Protein Expression. Cells. 2020 Jan 11;9(1).
